# Predictors of successful treatment in patients receiving intra-articular injections of hylan G-F 20 or saline for painful first MTPJ OA

**DOI:** 10.1186/1757-1146-4-S1-P42

**Published:** 2011-05-20

**Authors:** Shannon Munteanu, Gerard V Zammit, Hylton B Menz

**Affiliations:** 1 Department of Podiatry, Faculty of Health Sciences, La Trobe University, Melbourne, VIC, 3086, Australia; 2 Musculoskeletal Research Centre, Faculty of Health Sciences, La Trobe University, Melbourne, VIC, 3086, Australia

## Background

We recently conducted a placebo controlled clinical trial investigating the efficacy of Synvisc® (hylan G-F 20) for first metatarsophalangeal joint (MTPJ) osteoarthritis (OA). The aim of the current study was to devise a clinical prediction rule to identify those people with first MTPJ OA that are more likely to benefit from intra-articular injections (of hylan G-F 20 or saline).

## Methods

One hundred and fifty one participants with first MTPJ OA received an intra-articular injection of 1ml hylan G-F 20 or saline. Potential predictors included intervention group, demographics, height weight, BMI, symptom duration and severity, the Foot Health Status Questionnaire (FHSQ), first MTPJ dorsiflexion range of motion and radiographic OA severity (osteophytes and joint space narrowing as individual features in either the dorsal and lateral view, or as a combination, where scores of 2 for any feature in any view was considered ‘definite’ OA). The primary measure was a 15-point change in symptoms scale (range -7- ‘a very great deal worse’ to +7 – ‘a very great deal better’) at 12-weeks, dichotomised with scores of 3 defined as treatment success. Predictor variables were initially analysed with univariate analyses. Significant variables were than entered into a backward stepwise multivariate logistic regression.

## Results

At 12-weeks, 63 (42%) participants reported treatment success. Predictors of treatment success identified by univariate analyses were increased FHSQ foot function (mean difference 7.23, p=0.027), absence of definite radiographic OA (OR=2.49, p=0.017) and a score of 1 or less for osteophytes on the lateral radiographic view (OR=2.10, p=0.028). After multivariate logistic regression analysis, only FHSQ foot function (B=0.02, p=0.027, OR=1.02) and the absence of definite radiographic OA at the first MTPJ (B=0.906, p=0.017, OR=2.47) remained significant. The pre-test success rate of 42% increased to 57% at 12-weeks if the participant did not exhibit definite radiographic first MTPJ OA (positive likelihood ratio 1.86 (95% CI 1.11 to 3.13).

## Conclusions

This study identified few factors that could predict treatment success of intra-articular injections of hylan G-F 20 or saline for first MTPJ OA. Patients that do not display definite radiographic first MTPJ OA are more likely to report success of this treatment.

